# Coefficient corrections for portable X-ray fluorescence data of the Niton XL3t No. 97390 (coefcor I-IV) developed according to the Munich procedure

**DOI:** 10.1016/j.dib.2023.109914

**Published:** 2024-02-14

**Authors:** Michaela Schauer

**Affiliations:** a Department für Kulturwissenschaften, Geschwister-Scholl-Platz 1, Ludwig-Maximilians-Universität München, München 80539, Germany; b Universität Wien, Franz-Klein-Gasse 1, Vienna Institute for Archaeological Science (VIAS), Wien 1190, Austria; c Human Evolution and Archaeological Sciences (HEAS), Djerassiplatz 1, Universität Wien, Wien 1030, Austria

**Keywords:** p-XRF, Transparency, Quality control, Archaeology, Pottery, Correction factor, Linear regression, R

## Abstract

Portable X-ray fluorescence (p-XRF) devices are commonly utilized to analyze the chemical composition of various materials, such as archaeological pottery and siliceous substances. The discussion regarding the suitability of this method for such samples is ongoing, as the data are often said to be quantitatively unreliable. Nevertheless, the development of coefficient corrections (coefcors) offers a means to transparently demonstrate the quality and comparability of p-XRF data. These coefcors are established by comparing p-XRF measurements with laboratory methods derived from the same sample set. The Frankfurt Keramiklabor (Frankfurt Procedure) procedure serves as the basis for the Munich Procedure, which outlines criteria for assessing the quality of linear regressions applied to p-XRF data. The Munich Procedure provides clear benchmarks for the coefficient of determination (r²) and the relative standard error of the estimate (rSEE). Additionally, it includes a robustness test using a bootstrap method, as detailed in the R-scripts of this article. The Munich Procedure is applied to datasets generated using the Niton XL3t No. 97390 instrument used by the Dept. of Culture Sciences, Ludwig-Maximilians-Universität München (LMU), between 2017 and 2023. Measurements were conducted in the TestAllGeo mode, using an 8 mm collimator and a 300-second measurement time in air at Goethe-Universität Frankfurt. Data processing took place at LMU. Four datasets were utilized to develop four coefcors for comparing p-XRF and WD-XRF values of the Frankfurt pottery standard set (coefcor I-IV). Additionally, five coefcors were created to adjust p-XRF data from the same instrument before and after an instrument reset (coefcor ItoII, ItoIII, IItoIII, IIItoII, IVtoIII). The instrument provides analytical data, which is used to generate the dataset for developing coefficients using R-scripts. Processed data, including calculated criteria and factors (slope and intercept) for each chemical element, are presented with relevant graphical output. These coefcor factors can be utilized to correct empirical data obtained with Niton No. 97390 by selecting the appropriate coefcor based on the measurement date. This process is also outlined in the R-scripts. To assist users in applying the Munich Procedure to their own data, an R-script is provided that offers a more detailed explanation of the calculations. While the coefcors given here are specific to the instrument and therefore provide a basis for understanding the data quality of (as yet unpublished) studies using the Niton No. 97390, the method itself can be universally applied to develop coefcors for other p-XRF instruments.

Specifications Table

**Table:** 

Subject	Archaeology
Specific subject area	Coefcors for antique pottery/siliceous materials for p-XRFA
Data format	analytical data (.csv, .ndt), processed data (.csv), scripted (.qmd/.html/.pdf)
Type of data	table (.csv, .ndt), R-script (.qmd/.html/.pdf), figure (.eps)
Data collection	This dataset was collected between 2017 and 2022 using the Niton XL3t no. 97390, owned by the Dept. of Culture Studies, LMU. Selected samples from the Frankfurt standard set for ancient pottery were analysed using the TestAllGeo mode, an 8 mm collimator and 300 s measurement time. The sample set and the procedure for developing coefcors by comparing p-XRF values of these samples with laboratory values by calculating linear regressions in Excel was introduced to p-XRF studies of pottery in the German-speaking countries by Dr Markus Helfert (Frankfurt Procedure). His approach has been refined and further developed by the author and is presented the form of R scripts (Munich Procedure).
Data source location	Data collection: Goethe-Universität Frankfurt a. Main Forschungsstelle Keramik Norbert-Wollheim-Platz 1 60323 Frankfurt a. Main Germany Data ownership and storage: Ludwig-Maximilians-Universität Geschwister-Scholl-Platz 1 80539 München Germany
Data accessibility	Repository name: Open Data LMU Data identification number: 10.5282/ubm/data.405 Direct URL to data: https://data.ub.uni-muenchen.de/405/

## 1 Value of The Data

• By creating coefcors, the accuracy of p-XRF data is defined and comparability within and between p-XRF instruments, as well as with other chemical methods is obtained. However, coefcors generated by and used for p-XRF studies are still rare. This dataset may serve as a template for further publications of this kind.
• The coefcors provided here allow an assessment of the quality of the data obtained by p-XRF on Niton XL3t no. 97390 and thus provide the necessary transparency to classify the analyses and (archaeological or geochemical) interpretations of the data to which they have been applied.
• This data set can be used to compare the performance of the instrument presented here with other devices and methods. In this way, standards for high quality coefcors can be developed by the scientific community in the future.
• The code used to generate the coefcors is documented as R scripts and can therefore be applied by other scientists to their own data sets. The scripts can also be further developed, adapted and improved by the scientific community.

## 2 Data Description

This data set contains a total of nine coefcors for the Niton XL3t No. 97390 with all supporting material. The data provided is stored in four folders. Analytical data (data_analytical folder), representing the output of the instrument from the measurements taken to calculate each coefcor, is the basis for creating the actual coefcors. The term processed data (data_processed folder) then refers to the information generated by applying the Munich Procedure using R scripts (scripts folder) to the files stored in the analytical data folder. Graphics visualising important aspects of the so developed coefcors are also provided (graphics folder). Terminology and content of the data set and folders are explained in more detail in the following sections.

### 2.1 Relevance of Coefcors

Coefcors done by linear regressions are often applied to data collected by p-XRF instruments to compensate for matrix effects of the material being analysed, but also to assess the accuracy of the instrument in determining the agreement of its output with that of other analytical methods. Unlike calibrations, which also take into account elemental interferences as peak overlaps, coefcors consist of straightforward, direct comparisons of measurements made on the same set of samples with different settings, instruments or methods. In addition, coefcors can be used to align one device's values after a necessary recalibration done by the device manufacturer due to a defect of the device [1], [2], [3]. Thus, coefcors can be used to compare the performance of the same instrument before and after certain events, but also to correlate the measured values with those of the same sample determined by other analytical methods.

### 2.2 Terminology of the Coefcors of this Paper

**Fig. 1:**
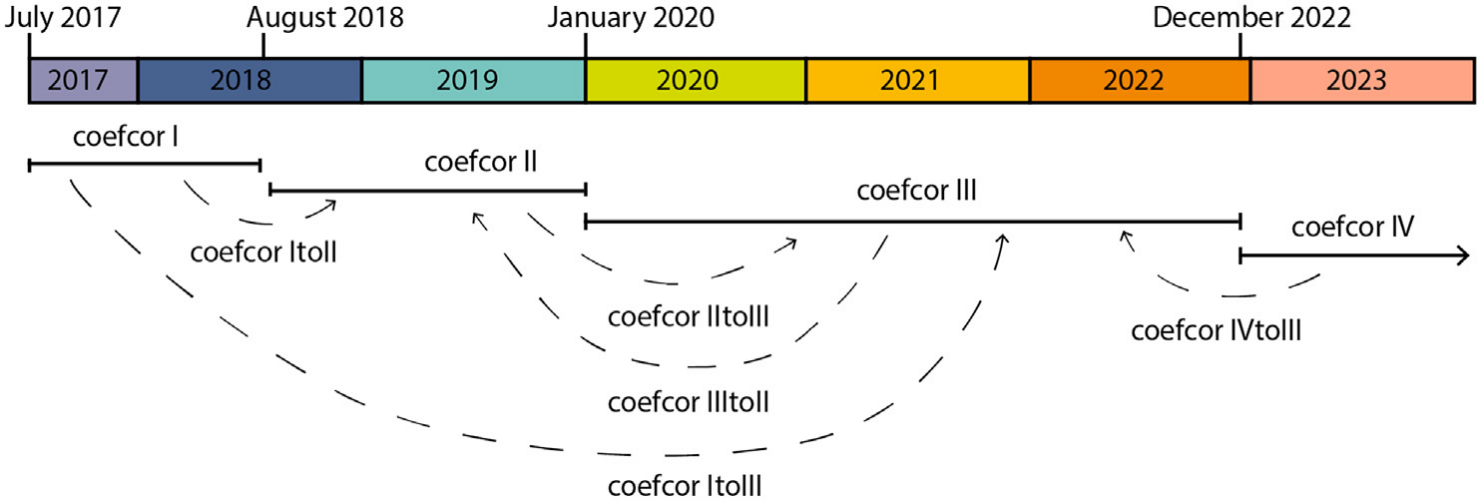
Timeline of the coefcors of this paper ((c) M. Schauer).

The coefcors presented here include both types mentioned above [4]. The coefcors based on comparisons of p-XRF analytical data and laboratory method values are referred to as coefcor I to IV. Each of these coefcors apply to data collected in a specific timeframe by the Niton XL3t no. 97390 (Fig. 1):

- coefcor I: data collected between July 2017 and August 2018
- coefcor II: data collected between September 2018 and January 2020
- coefcor III: data collected between January 2020 and December 2022
- coefecor IV: data collected since December 2022

Coefcors for in-device corrections have been created specifically for archaeological projects where measurements were taken before and after an instrument reset. In these cases, the coefcors are given the number of the coefcor that was valid in the period of the data to be fitted, followed by a ‘to’ and the name of the coefcor that was used in the period to be adjusted to. For example, if the first measurements of a project started in July 2019, but the majority of the samples were taken in May 2021, it makes sense to adjust the data from 2019 (coefcor II period) to those from 2021 (coefcor III period). In this case, the corresponding coefcor will be labelled coefcor IItoIII. Due to the need to interpret the (unpublished) archaeological projects of the LMU, this dataset contains coefcor ItoIII, coefcor IItoIII, coefcor IIItoII and coefcor IVtoIII.

### 2.3 Analytical Data (Folder Data_Analytical)

**Fig. 2:**
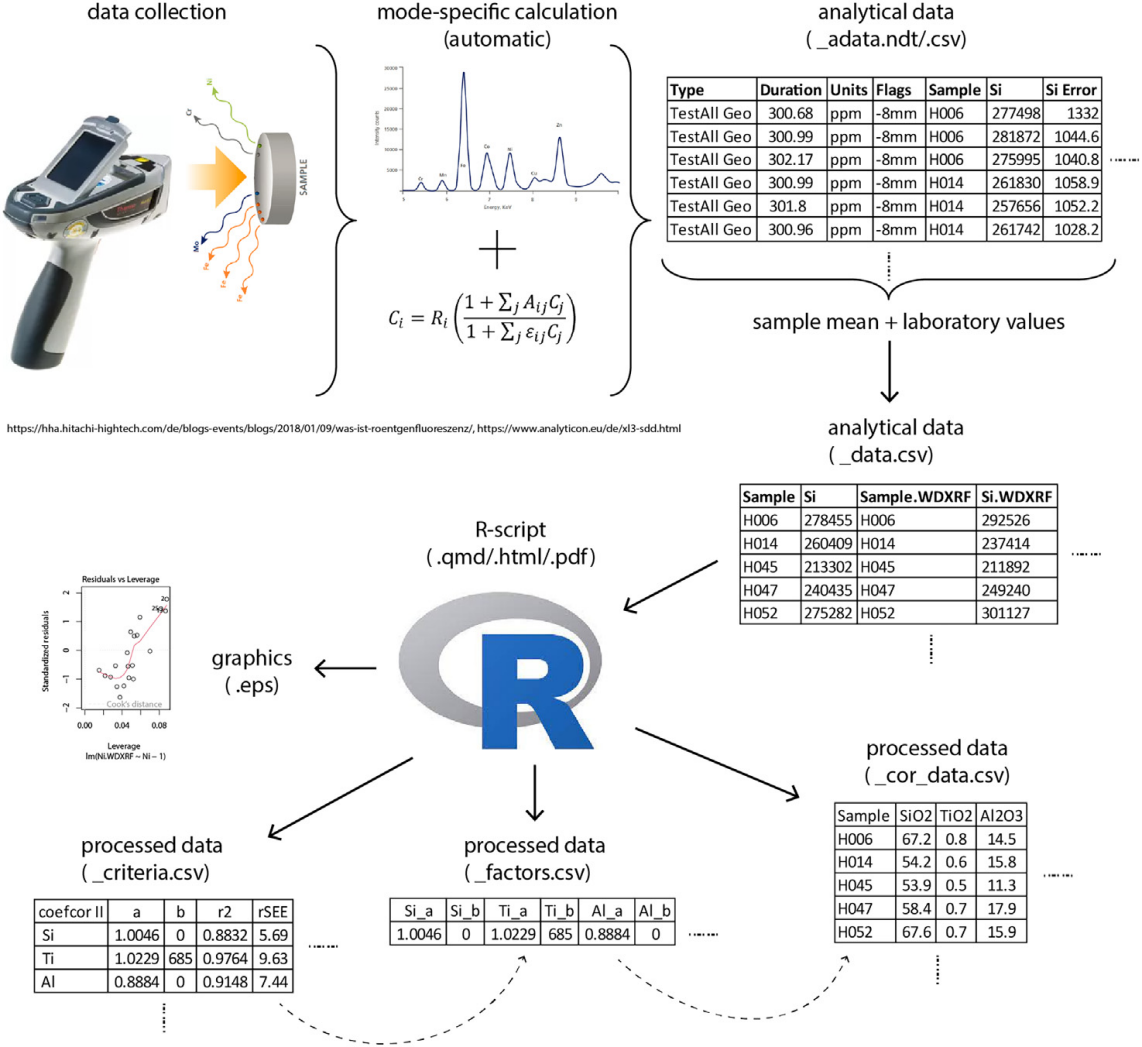
Terminology and data processes ((c) M. Schauer).

With regard to Niton instruments, the data used to generate the coefficients in this paper are not raw data in the strict sense: The files produced by the instrument contain values calculated by the selected measurement mode (Fig. 2). However, there is no output of raw counts or intensities (raw data). Accordingly, it is more appropriate to refer to the instrument's output in the form of a spreadsheet as analytical data. These tables - coded with the extension ‘adata.ndt^1^/.csv’ (e.g., coefcorI_adata.ndt/.csv) - are stored in the ‘data_analytical’ folder in the folder with the name of the respective coefcor (e.g., coefcorI). These files contain technical information, metadata and measurement data. The former consist of Reading-No (the internal measurement no automatically given by the instrument), Time (of measurement), Type (measurement mode), Duration (measurement time), Units (in this case ppm), Sigma Value (related to the standard deviation given for each measurement, here 2σ) and Flags (collimator size). Metadata is given in the Sample column (name of the sample measured). This is followed by the measured value for each element (named Sifor example) with the standard deviation given in the next column (named Si Error for example) and the count rate – here presented with 0-values (names Si CPS/ua for example). The order of the chemical elements follows that of the .ndt files.

To work with the p-XRF data for each sample, the average of up to three measurements is calculated and transferred to a new .csv spreadsheet, also stored in the ‘data_analytical’ folder in the respective coefcor folder. Only the columns containing sample number and chemical element values are included, therefore these files are called _data.csv (e.g. coefcorI_data.csv). The elements are arranged according to the standards of geological chemical analysis. As a special feature of the Niton instrument, information on the so-called balance is given in the last columns. This value represents the matrix fraction calculated from the scattered radiation generated during the measurement in the standard filter. It is considered as a proxy for the components of the sample that cannot be measured by p-XRF (e.g. oxygen, carbon, etc.). This allows a more accurate quantification of the concentration of the elements that can actually be measured by the method [[1], [2], [3], [5]]. Following these columns, the correlating values for each sample and element generated by a laboratory method –in this case WD-XRF - are added using the information provided in the spreadsheet MHelfert_FrankfurtMethod_WDXRFdata.csv which is located in the ‘data_analytical’ folder. The column headings for this dataset are identified by adding an abbreviation of the method to the header. This is necessary so that R can distinguish between the columns relating to p-XRF measurements (e.g. Si) and those relating to the laboratory method (e.g. Si.WDXRF). All measured values are unnormalised (i.e. not recalculated to 100% [3]) and expressed in ppm. This table therefore consists of the analytical data used to develop the coefficients.

### 2.4 R-Scripts (folder scripts), Processed Data (folder data_processed)

To facilitate the handling of data and scripts, the documentation is organized as an R-project. The R project file, coefficient_corrections.rproj, is located in the ‘coefficient corrections’ folder. This serves as the starting point for working with R. When opened, the file contains all the R scripts referred to in this publication. These executable scripts are provided for each coefcor and are stored as .qmd, .html, and .pdf files in the ‘scripts’ folder contained within the respective coefcor folder. They encompass all the necessary calculations of the Munich Procedure (described below) and include graphical output when applicable. The script calculation_coefcor_example.qmd/.html/.pdf offers a step-by-step introduction to the Munich Procedure and provides additional explanations of the code. All other scripts present the calculations for each coefcor and are named accordingly (e.g. coefcorI.qmd/.html/.pdf). The relevant coefcor criteria (e.g. slope, r², rSEE, etc.; see below) are documented as processed data in the _criteria.csv spreadsheets (e.g. coefcorI_criteria.csv). These spreadsheets are stored in the ‘data_processed’ folder within the respective coefcor folder. Additionally, the slope and intercept information for each chemical element is exported into a separate file named _factors.csv (e.g. coefcorI_factors.csv). This file serves as the basis for correction calculations of empirical p-XRF data (e.g. measurements of archaeological artifacts, soils, etc.) and is stored in the same folder. Subsequently, this file is used to perform the coefcor on the _data.csv file, which was initially used to generate these factors (e.g. coefcorI_data). The results of these calculations are presented in the _cor_data.csv files (e.g. coefcorI_cor_data.csv) and are also stored in the ‘data_processed’ folder of the respective coefcor (e.g. coefcorI).

### 2.5 Graphics (folder graphics)

The graphics (.eps)^2^ for each coefcor are located in a ‘graphics’ folder within the coefcor folder(s). For each chemical element, all relevant plots of RTO and OLR (see below) are exported as a file with the name of the coefcor, the element and the type of linear regression, ending with the suffix _allfig.eps (e.g. coefcorI_Al_OLR_allfig.eps). In addition, all scatterplots from p-XRF to WD-XRF values of the selected linear regression for each element are exported together in the file _traditional_scatterplots.eps (e.g. coefcorI_traditional_scatterplots). In rare cases, the residuals versus leverage plots had to be exported individually. These are named after the type of linear regression and the chemical element with a ‘5’ added (e.g. RTOSi5.eps). The latter is derived from the plot number when exporting descriptive graphs for linear regressions. They are located in the graphics folder of the respective coefcor.

## 3 Experimental Design, Materials and Methods

### 3.1 Data Collection

The data presented here were collected by Helfert, Schauer and Maschulat between 2017 and 2022 at the facilities of the Forschungsstelle Keramik at the Goethe-University Frankfurt using the Niton XL3t no. 97390 in the TestAllGeo-mode. The main filter thereby covers most of the relevant trace elements (antimony, arsenic, bismuth, cadmium, cobalt, copper, lead, molybdenum, nickel, niobium, palladium, rubidium, selenium, silver, strontium, thorium, tin, tungsten, uranium, zinc and zirconium) as well as the main elements iron and manganese. The following low filter measures the elements chromium, vanadium and titanium, while the high filter concentrates only on barium. The light filter gives the concentrations for aluminium, chlorine, potassium, calcium, magnesium, phosphorus, sulphur and silicium. This mode uses both Compton peak normalisation and a fundamental parameter algorithm to calculate concentrations [1,3].

Measurements were taken at ambient temperature, with the instrument unevacuated and positioned on its side. Objects were arranged as close and parallel as possible [3,5,6] to the measuring window with the aid of organic matter, and the window was protected by 4µm Mylar film [7]. Temperature (18 to 28 °C) and relative air humidity (55–75%) were, to the best of our knowledge, within acceptable limits at the time of the measurements [8]. Main, low and high filter of the TestAllGeo-mode were set to 60 s and the light filter to 120 s, giving a total measurement time of 300 s [1]. This approach follows the Frankfurt Procedure and is supported by runtime tests performed with the Munich instrument. As recommended for pottery analysis, an 8 mm collimator was used [9] and each sample was measured at least once, but whenever possible in three non-overlapping areas of a fresh brake [1,3,5]. A mean value was then calculated for each sample and used in the coefcor [3].

### 3.2 Data Processing

The coefcors presented here were created following the Munich Procedure (see below) using Rgui (version 4.3.1) for code development and RStudio (version 2023.06.1 – Build 524) with the Quarto plugin (version 1.3.433) for scripting.

### 3.3 The Frankfurt Procedure

The use of coefficients for p-XRF studies of ancient pottery was introduced to archaeometric studies in the German-speaking countries by Dr Markus Helfert and is now widely used, particularly by specialists using Niton instruments [1,[10], [11], [12]]: Helfert uses archaeological pottery samples (Frankfurt Pottery Sample Set) to develop a linear regression to compare values measured by p-XRF (x-values or independent variable) with values of the same sample set obtained by a laboratory method - in this case WD-XRF (y-values or dependent variable) [1,3,13]. The slope (a) and, if appropriate, the intercept (b) are then used as coefficients to correct the p-XRF values. The exclusion of samples from the calculation in order to obtain the best possible equation is done individually, based on the operator's judgement and the visible change in the values of the coefficients and the quality criterion of the coefficient of determination (r²). Applying the coefficients should introduce as little change to the original p-XRF-values as possible and r² should be close to 1. The extent of the mathematical corrections applied and the value of r² then provide the basis for deciding whether a regression trough origin (RTO) or an ordinary linear regression (OLR) should be used [14,15].

### 3.4 The Munich Procedure

The author of this paper was trained by Helfert and has developed the Frankfurt Procedure further by

(a) switching from Excel to R in 2021, thus providing a scripted method
(b) providing a direct and detailed comparison between RTO and OLR
(c) adding additional quality criteria for evaluating the linear regressions
(d) defining clear criteria for selecting the best linear regression
(e) compiling the defining criteria of the chosen linear regression in a standardized, comprehensible and transparent manner

The first step of the Munich Procedure is to export the required tables from the manufacturer's NDT program using the .ndt files of the respective coefcor (see above). Various settings can be made in the NDT-program. The author has chosen to retain the information when measurements are below the Limit Of Detection (LOD) and to display the count rate so that no further calculations are done performed by the program during the export. ‘LOD’ has subsequently been replaced by ‘0’ in the _adata.csv files.

The procedure for creating a coefcor from the data.csv files in R starts with a comparison of all value pairs of the provided dataset, where OLR and RTO are calculated [14,15]. Relevant quality criteria for estimating these regressions are r² and the standard error of the estimate or residual standard error (SEE) [15,16]. The simple SEE thereby describes the 1σ confidence interval, while the ± 1.5-fold SEE describes the 2σ confidence interval [16]. Here the former SEE is used. In addition, the root mean squared error (RMS) could be considered to support the conclusions drawn from the SEE: While the latter indicates the average distance of the observed values from the regression line, the former describes the deviation of the values in the unit of measurement from their actual ‘correct’ position (e.g. the value of the laboratory method) [15]. To ensure comparability of the estimation error of each element and between OLR and RTO, the relative SEE (rSEE) is calculated using the formula: rSEE=(SEE/mean)*100. The thresholds for both criteria named above are defined by the author as r²≥0.9 and rSEE≤10%. It should be noted that when working with RTO, Excel automatically adjusts the calculation of r² to the required conditions, whereas R does not. Here, this adjustment must be made manually in order to make a direct comparison [14,17].

To optimize the coefcors, significant outliers (o') are excluded based on the analysis of the residuals. Looking at the QQ plot, where the line formed by the residuals should be as straight as possible, gives a good first impression. However, in order to make a clear decision as to which of the potentially conspicuous samples are actually significant outliers, a Bonferroni outlier test is performed [15]. Samples are then removed for the next data run if their ρ-value is significant. Therefore, to accept a coefcor, the criterion of $\rho_{o^{\prime }}>0.05$ [15,16,18] must be met for the chosen linear regression. Additionally, the outlier of both regressions should be different samples: $o{{}^{\prime }}_{OLR}\neq \;o{{}^{\prime }}_{RTO}$. The latter criterion can be disregarded if the other criteria are met well enough. Note that if removing an outlier worsens the values of r² and/or rSEE, either the outlier from the other linear regression, or an outlier identified on the basis of the QQ plots relevant to the better or both linear regressions is removed.

In order for a coefficient to be accepted, it must also pass a test for the reproducibility of the slope estimate using the bootstrap method. The bootstrap confidence intervals are calculated using the BCa (bias-corrected and accelerated) approach based on the actual values of the cases drawn (case resampling) with a confidence level of 0.9 (90%) and drawing 2500 random samples [16,19,20]. To pass this test, the values obtained by the bootstrap method (Bs) and those calculated on the basis of the linear regression (LR) must correspond as closely as possible to the 5% and 95% confidence levels (CI 5, CI 95). In this context, deviations of more than 0.05 are considered to be too high. The benchmark to be met can therefore be expressed as $CI_{LR_{5,95}}=CI_{Bs_{5,95}}\pm \;0.05$.

In practice, the scatter plot as well as the correlation of the unadjusted data pairs also give a good idea of how complex the creation of a robust coefcor will be, because the higher the correlation, the fewer outliers need to be removed. In addition, the QQ plot was particularly helpful in assessing whether removing the outliers generated by the Bonferroni test - regardless of their significance - would result in a significant improvement in the linear regression. The reproducibility of the calibration was expressed mainly in the agreement of the expected values of the confidence levels with those of the bootstrap. Thus, some calibrations were found to be very robust even if they had an r² value below 0.9 or an rSEE above 10%, so that even elements for which one of these limits was not met were accepted. As a general rule, it should be noted that slope correction factors that differ greatly from 1 always carry a higher risk of mis-estimation and should therefore be used with greater caution. The same applies if more than 35% of the original value pairs had to be removed to obtain an acceptable coefficient correction.

In summary, according to the Munich Procedure, the decision as to whether OLR or RTO is more appropriate for the particular element is mainly based on the comparison of the values of r², rSEE and the result of the bootstrap procedure. The final decision on whether to use RTO or OLR for a particular element is made when either or both regressions meet all of the benchmarks described above. In addition to these, the range of variation of the residuals, the percentage of excluded samples and the value of the RMS should also be considered. All these factors can be used to compare coefcors and to define and document their quality in a transparent way.

### 3.5 Applying Coefcors to Empirical Data

To apply coefcors to empirical data sets, the appropriate coefcor for the year of analysis must first be selected (see also Fig. 1). Then the slope and intercept corrections are applied individually for each element and each measurement. This process is automated using the R scripts of the Munich Procedure. When working with internal coefcors (e.g. coefcorItoII), the major elements are not converted to oxide percentages. Their normalisation to 100% is also omitted. When using coefcors I to IV, these steps are included.

## Limitations

The specific coefcors developed here, and the values of their criteria and factors, are only valid and can only be applied to data generated by the Niton XL3t No 97390 in the time period associated with each coefcor. However, the Munich Procedure as a process can be used to develop coefcors independently of the manufacturer, instrument or underlying data set.

## Ethics Statement

The author has read and followed the ethical requirements for publication in Data in Brief and confirms that the current work does not involve human subjects, animal experiments, or any data collected from social media platforms.

## CRediT authorship contribution statement

**Michaela Schauer:** Funding acquisition, Conceptualization, Methodology, Software, Formal analysis, Data curation, Writing – original draft.

## Data Availability

R-scripts and data of coefficient corrections developed since 2017 for the Niton XL3t No. 97390 following the Munich Procedure (Original data) (Open Data LMU).

## Footnotes

1 The .ndt files are the device's own format which can only be opened with a specific programme provided by the manufacturer.

2 This decision was made to keep the file size as small as possible to facilitate reuse and easy downloading. If other formats are required - the graphics can be saved as .jpg files from the html files of the corresponding coefcor.
